# *Limosilactobacillus reuteri* - a probiotic gut commensal with contextual impact on immunity

**DOI:** 10.1080/19490976.2025.2451088

**Published:** 2025-01-17

**Authors:** Amanda H. Lee, Daphne Michelle Rodriguez Jimenez, Marlies Meisel

**Affiliations:** a Department of Immunology, University of Pittsburgh School of Medicine, Pittsburgh, PA, USA; b Graduate Program of Microbiology and Immunology, University of Pittsburgh School of Medicine, Pittsburgh, PA, USA; c Cancer Immunology and Immunotherapy Program, UPMC Hillman Cancer Center, Pittsburgh, PA, USA

**Keywords:** Autoimmunity, cancer, immune system, Limosilactobacillus reuteri, probiotic, systemic disease

## Abstract

The gut microbiome plays a key role in human health, influencing various biological processes and disease outcomes. The historical roots of probiotics are traced back to Nobel Laureate Élie Metchnikoff, who linked the longevity of Bulgarian villagers to their consumption of sour milk fermented by Lactobacilli. His pioneering work led to the global recognition of probiotics as beneficial supplements, now a multibillion-dollar industry. Modern probiotics have been extensively studied for their immunomodulatory effects. *Limosilactobacillus reuteri* (*L. reuteri*), a widely used probiotic, has garnered significant attention for its systemic immune-regulatory properties, particularly in relation to autoimmunity and cancer. This review delves into the role of L. reuteri in modulating immune responses, with a focus on its impact on systemic diseases.

## Introduction

### Probiotics definition and historical background

The Nobel Laureate Ilya Ilyich (Élie) Metchnikoff, celebrated as the father of innate immunity for his discovery of phagocytosis, later earned another notable title: the “Father of Probiotics”.^1^ While studying the remarkable longevity of residents in a region of Bulgaria, Metchnikoff hypothesized that their advanced age was linked to their diet, particularly their consumption of sour milk.^2^ He advocated for daily consumption of milk fermented with lactic acid bacteria as a cornerstone of a long and healthy life. When Metchnikoff’s theory made headlines in Paris in 1899, it sparked a surge in demand for yogurt, popularizing probiotics to the masses. Today, these lactic acid bacteria, now termed “probiotic bacteria,” are among the most widely consumed dietary supplements globally.^1^ The Food and Agriculture Organization of the United Nations (FAO) and World Health Organization (WHO) define probiotics as “live microorganisms that, when administered in adequate amounts, confer a health benefit on the host”.^3^ The probiotic industry has since evolved into a multibillion-dollar market,^4^ underscoring a need to investigate the impact of probiotics on human health.

### Application value and potential of probiotics

Probiotics hold significant application value and potential in promoting human health and managing diseases. Probiotics have shown promise in preventing and treating gastrointestinal disorders, such as irritable bowel syndrome (IBS),^5^ inflammatory bowel disease (IBD),^6^ and antibiotic-associated diarrhea.^7^ Beyond gut health, probiotics influence the immune system, reduce inflammation,^8^ and impact mental health via the gut-brain axis.^9^ Emerging evidence links probiotics to chronic conditions like obesity,^10^ diabetes,^11^ cardiovascular diseases,^12^ and cancer^13^ by modulating host-microbe interactions and enhancing immune responses. With over 1,000 clinical trials as of 2020, probiotics are highly of interest for therapeutic use.^14^

### *Limosilactobacillus reuteri* as a model for studying probiotic-driven immunomodulation

To fully harness the potential of probiotics, it is critical to understand the role of specific commensals and the mechanisms they employ. *Limosilactobacillus reuteri* (*L. reuteri*) is one of the most frequently used and well-studied probiotics, with decades of research highlighting its immunomodulatory properties.^15–18^ Due to the co-evolution of *L. reuteri* with its hosts, there is significant genetic heterogeneity within *L. reuteri* populations and consequently many strain-specific and context-dependent effects that offer key insights into leveraging probiotics to shape human health.^19,20^

In this review, we will almost exclusively focus on the systemic immunoregulatory impact of *L. reuteri* during disease, especially in autoimmunity and cancer. We briefly discuss the impact of *L. reuteri* on mucosal immunity and gastrointestinal pathology to provide a foundation for exploring its broader systemic impacts on immunity, but this topic is addressed more in depth by others.^4,15,21–23^

## *Limosilactobacillus reuteri’s* impact on its niche

### A brief historical background

*L. reuteri* was first isolated by and named after German microbiologist Gerhard Reuter in 1962.^24,25^ Reuter discovered that *L. reuteri* was native to the human microbiome, with significant levels present within the small intestine and feces of healthy individuals. Controversial at the time, Reuter also found significant levels of *Lactobacilli* in both the stomach and duodenum, areas previously considered sterile sites of the gastrointestinal tract (GIT).^26^ *L. reuteri* is a Gram-positive, nonsporulating, facultative anaerobic species of *Limosilactobacillus* that can thrive in a wide variety of environments. *L. reuteri’*s persistence in the host can be partially attributable to its resilience to the low pH and bile salts of the GIT,^27,28^ especially in the small intestine – the main niche of *L. reuteri*.

### Role of *L.* *reuteri* in maintaining intestinal barrier integrity

The intestinal mucosal barrier is a critical immune defense barrier where trillions of microbes and environmental antigens interact with host immune cells, making it a key site for maintaining immune homeostasis.^29^ Intestinal barrier defects can decrease immune tolerance to environmental antigens and allow for the spread of pathogenic bacteria (“leaky gut”).^30^ Disruption of the intestinal barrier is implicated in several disease states, especially autoimmune and inflammatory disorders.^29,31–33^ *L. reuteri* is involved in the development and differentiation of intestinal epithelial cells (IECs), which modulate the immune system to protect immune homeostasis.^34^ Co-culture of *L. reuteri* D8 with intestinal organoids increased the proliferation of leucine rich repeat containing G protein-coupled receptor 5 (Lgr5) positive intestinal epithelial stem cells (ISCs) through activation of the Wnt/β-catenin pathway, promoting the differentiation of antimicrobial peptide-secreting *Paneth* cells.^22^ *L. reuteri* can act defensively on the intestinal barrier, preventing the colonization of pathogenic bacteria by producing anti-microbial peptides or competing for resources. *In vivo*, the *L. reuteri*-mediated protection from *Citrobacter rodentium* infection was linked with an increase in Lgr5^+^ ISC proliferation and expansion of lysozyme^+^ *Paneth* cells.^35^ Furthermore, *L. reuteri* has been implicated in the production of short-chain fatty acids (SCFAs), such as butyrate, and aryl hydrocarbon receptor (AhR) ligands, both of which have been associated with improved intestinal barrier function.^36–39^ A study found that *L. reuteri*-mediated enhanced AhR agonism reduced fecal levels of lipocalin-2 and paracellular permeability. Notably, these changes were associated with decreased immunopathology in autoimmune prone nonobese diabetic (NOD) mice expressing DQ8, a celiac disease susceptibility gene,^40^ suggesting that the protection of intestinal barrier integrity may be one mechanism by which *L. reuteri* protects against the development of autoimmune and inflammatory diseases.

In addition to its increase of physical barrier protections, *L. reuteri* demonstrates immunomodulatory properties that play a role in both fortifying tight junctions (TJs) in the intestinal barrier and regulating mucosal immune cell function. Administration of *L. reuteri* I5007 to newborn piglets led to an overall increase in the protein expression of claudin-1, occludin, and zonulin-1 in IECs.^41^ *In vitro* studies utilizing IPEC-J2 intestinal porcine enterocytes demonstrated that treatment with *L. reuteri* I5007 maintained intestinal barrier integrity.^42^ Treatment with *L. reuteri* I5007 supernatant was sufficient to reverse lipopolysaccharide (LPS)-induced increase of proinflammatory cytokines tumor necrosis factor-α (TNF-α) and interleukin-6 (IL-6) and decrease of TJ proteins.^42^

### *L.* *reuteri* maintains intestinal immunological homeostasis in its niche

**Figure 1.**
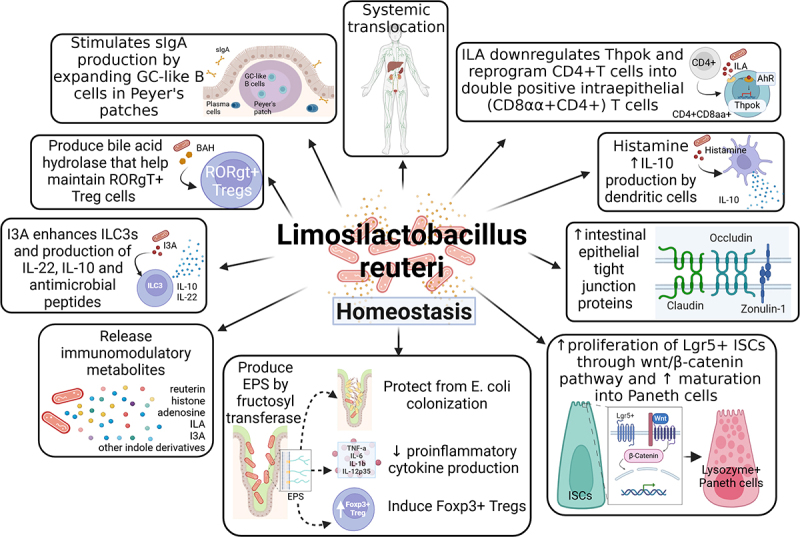
Role of *Limosilactobacillus reuteri* in the context of intestinal and immune homeostasis. During homeostasis, *L. reuteri* strengthens the intestinal barrier by increasing intestinal epithelial tight junction protein expression, producing antimicrobial peptides, binding to intestinal epithelial cells via exopolysaccharides, increasing maturation of lysozyme secreting *Paneth* cells and releasing immunomodulatory metabolites. Specifically, ILA downregulates Thpok, which then reprograms CD4+ T cells into double positive intraepithelial CD8αα+CD4+ T cells. Additionally, I3A enhances ILC3s and the production of IL-22, IL-10 and antimicrobial peptides. *L. reuteri* suppresses intestinal inflammation via secretion of secretory IgA, maintenance of RORgT+ regulatory T cells by producing bile acid hydrolase, induction of Foxp3+ regulatory T cells, reduction of proinflammatory cytokines (e.g. IL-6, TNF-α, IL-1β), and increase of IL-10-producing myeloid plasmacytoid dendritic cells. *L. reuteri* and other commensals can translocate to systemic sites and various organs including liver, spleen, and extraintestinal tumors during homeostasis or disease-state. The summarized characteristics of this figure should not be considered as common characteristics of this species but rather possible functions of the species in context of homeostasis and systemic diseases.

Legend: AhR, aryl hydrocarbon receptor; BAH, bile acid hydrolase; DC, dendritic cell; EPS, exopolysaccharide; Foxp3, Forkhead box protein P3; I3A, indole-3-aldehyde; IL, interleukin; ILA, indole-3-lactic acid; ILC, innate lymphoid cell; ISC, intestinal stem cell; Lrg5, leucine-rich repeat containing G-protein-coupled receptor 5; RORgT, RAR-related orphan receptor gamma; sIgA, secretory immunoglobin A; Tet, tet methylcytosine dioxygenase; TH, T-helper; TNF-α, tumor necrosis factor alpha; Treg, regulatory T cell.

Several mechanisms of how *L. reuteri* maintains immunological tolerance to protect from intestinal inflammation have been reported (Table 1 and Figure 1). Many of the metabolites produced by *L. reuteri* to promote its survival and colonization also benefit the host by suppressing excessive inflammation and pathogenic bacteria. Exopolysaccharides (EPS) produced by *L. reuteri* strengthens its adhesion to IECs, protecting from colonization of pathogenic bacteria, such as *E. coli* .^80^ Additionally, EPS can suppress intestinal inflammation either directly through (i) the suppression of cytokine function, or (ii) the induction of forkhead box P3 (Foxp3)^+^ regulatory T cells (Tregs). EPS co-culture with IPEC-J2 epithelial cells significantly decreased the production of pro-inflammatory cytokines TNF-α, IL-6, IL-1β, and IL-12p35.^80^ EPS derived from *L. reuteri* 100–23 also served to dampen inflammation by increasing the number of Treg cells in the spleen of rodents. Importantly, the ability of *L. reuteri* to enhance systemic Treg expansion depends on its production of EPS by fructosyl transferase.^56^ *L. reuteri* is also able to produce bile salt hydrolase (BSH), necessary for its survival in environments containing high levels of bile salts.^81^ In BSH mutant mice lacking two key BSH genes (BT_1259 and BT_2086), the number of colonic retinoic acid receptor-related orphan receptor gamma t (RORγt) positive Treg cells were significantly reduced due to a Treg intrinsic upregulation of the bile acid sensing vitamin D receptor (*Vdr*).^82^ As RORγt^+^ Tregs help maintain colonic homeostasis,^83^ BSH produced by *L. reuteri* and other commensals likely contribute to maintaining tolerance.^82^

**Table 1.** Impact of *L. reuteri* colonization during homeostasis, autoimmunity, and cancer.

Strain of *L. Reuteri*	Ref.	Species	Condition/ treatment	Duration of treatment	Model	Pathology	*In vivo* effects	Effect on disease severity	Effects on gut microbiome	Mechanisms	Comments
**Human Trails**											
DSM 17,938/ATCC PTA 5289	^43^	Human	Orally administered 2 tablets twice daily	3 weeks	Healthy individuals	Homeostasis	No change in salivary secretory IgA or cytokine levels	N/A	N/A	N/A	N/A
DSM 17,938/ATCC PTA 5289	^44^	Human	Orally administered 1×10^9 CFU/tablet twice daily	12 weeks	Healthy individuals	Homeostasis	Increased salivary IgA protein but no differences in cytokine levels	N/A	N/A	N/A	N/A
ATCC PTA 5289/ATCC 55,730	^45^	Human	Orally administered 2×10^8 CFU/gum 10 minutes twice daily	12 weeks	Healthy individuals	Homeostasis	Increased salivary IgA protein; *S. mutans* 10449 and *S. sobrinus* B13 specific antibodies	N/A	N/A	N/A	N/A
RC14	^46^	Human	Orally administered 10^3 CFU/mL 125 g yogurt daily	30 days	IBD patients	Autoimmunity	Blood: Increased proportion of CD4+ CD25+ Treg cells in peripheral blood; Serum: Decreased percentage of IL-12 and TNF-α producing monocytes and myeloid dendritic cells; decreased IL-12 concentration	Decrease	N/A	N/A	Given in combination with *L. rhamnosus*
ATCC 55,730	^47^	Human	Rectal enema 10^10 CFU daily	8 weeks	IBD patients	Autoimmunity	Increased IL-10 and decreased IL-1β, TNF-α and IL-8 expression levels in rectal mucosa	Decrease	N/A	N/A	N/A
RC14	^48^	Human	Orally administered 2×10^9 CFU total twice daily	3 months	Patients with RA	Autoimmunity	Serum: Decreased levels of IL-1α, IL-6, IL-10, IL-12p70, TNF-α and MCP1; Clinical: no clinical improvement but had functional/wellbeing improvements	no clinical improvement	N/A	N/A	N/A
PBS 072	^49^	Human	Orally administered 1×10^9 CFU/tablet daily	56 days	Individuals with AD	Autoimmunity	Improved skin smoothness, moisturization and self-perception; decreased inflammatory markers TNF-α, TARC and TSLP	Decrease	N/A	N/A	Given in combination with *L. rhamnosus*, *L. plantarum*
DSM 12,246	^50,51^	Human	Orally administered 10^10 CFU dissolved in liquid twice daily	6 weeks	Individuals with AD	Autoimmunity	Decreased severity of eczema by 4.5% with more pronounced effects in patients with high IgE levels; decreased small intestinal permeability	Decrease	N/A	N/A	Given in combination with *L. rhamnosus* 19070–2
**Animal Models**											
I5007	^52^	Pig	Sows: 10^9 CFU/kg diet Piglets: oral gavage 5 mLs of 10^9 CFU/mL	Sows: supplement diet for 28 days Piglets: oral gavage at birth	Landrace-Yorkshire sows and piglets	Homeostasis	Sows: dietary supplementation increased lactate concentration in colostrum and TNF-α and IL-6 in cord blood serum Piglets: oral gavage decreased serum IL-6 in piglet	N/A	Maternal dietary supplementation increased alpha diversity in meconium of piglets; increased *Romboutsia*, *Lactobacillus*, *Blautia*, *Butyricicoccus* and *Ruminococcus* Oral gavage increased alpha diversity and abundance in *Clostridium*, *Blautia*, *Lactobacillus*, and *Ruminococus*; decreased abundance of *Escherichia/Shigella* and *Enterococcus* families in fecal samples of piglets	N/A	N/A
I5007	^41^	Pig Cells	Piglets: oral gavage 6 × 10^9 CFU Cells: 3 × 10^7 CFU/mL	Piglets: 14 days Cells: 0–10 hours	Landrace-Yorkshire piglets/ Intestinal porcine enterocyte (IPEC-J2) cell line	Homeostasis	Increased abundance of claudin-1, occludin and ZO-1 proteins in the Ileum and of occludin and ZO-1 in jejunum	N/A	N/A	Increased expression of tight junction proteins such as claudin-1, occludin and ZO-1 protecting from gut permeability	N/A
I5007	^42^	Pig Cells	Piglets: oral gavage 1 × 10^10 CFU Cells: 3 × 10^7 CFU/mL	Piglets: 20 days Cells: 3–12 hours	Piglets Intestinal porcine enterocyte (IPEC-J2) cell line	Homeostasis	Increased *pBD2*, *pBD3*, *pBD114*, *pBD129* and *PG1–5*	N/A	Increased *Paraprevotellaceae* and *Sharpea*	Increased mRNA expression of β-defensins (*pBD2*, *pBD3*, *pBD114*, *pBD129* and *PG1–5*) which act as antimicrobial peptides	N/A
22	^53^	Chicken	Oral gavage 10^8 CFU	7 days	Chickens	Homeostasis	Increased intestinal mRNA expression of *Lrg5*, *Axin2* and *Lrp5*, activated Wnt/β-catenin signaling pathway and induced proliferation; inhibited Notch signaling pathway by decreased (although not significantly) *DLL1*, *Notch1* and *Hes1* expression and increased *Muc2* expression	N/A	N/A	Induced intestinal proliferation by the activation of the Wnt/β-catenin (*Lrg5*, *Lrp5* and *Axin2*) signaling pathway	N/A
PSC 102	^54^	Rats Cells	Rats: Oral gavage 10^9 CFU/mL Cells: 10^9 CFU/mL	3 weeks 24 hours	Cyclophosphamide-induced immune-suppress Sprague-Dawley rats murine macrophage Raw264.7 cell line	Homeostasis	Increased neutrophil migration, phagocytosis, splenocyte proliferation and CD45RA+ T cells; upregulated IL-2, IL-4, IL-6, IL-10, IL-12A, TNF-α and IFN-γ in serum	N/A	Increased intestinal microbiota richness, relative abundance of *Prevotella* and *Oscillibacter* and reduced *Fusobacterium* and *Bacteroides*	N/A	N/A
222/1021	^55^	Rats	Rats oral gavage 10^9 CFU daily	4 weeks	Sprague-Dawley rats	Homeostasis	Increased fecal free sIgA in a vitamin A dependent manner	N/A	Increased gut microbiota diversity in small intestine	N/A	N/A
DSM 17,509	^56^	Mice	Oral gavage 10^6 CFU g body wt −1 daily	Once	Lactobacillus-free BALB/c mice	Homeostasis	Produced exopolysaccharide levan through FTF action; induced Foxp3+ Treg cell populations in spleen	N/A	N/A	N/A	N/A
L7(WU)/100–23	^57^	Mice	Oral gavage 1×10^9 CFU/mL daily	2 days	C57BL/6 GF or WT mice	Homeostasis	Produced derivatives from dietary tryptophan, such as ILA, that activated AhR and downregulated Thpok which reprogram CD4+ T cells into double positive intraepithelial lymphocytes (CD8aa+ CD4+ T cells)	N/A	N/A	N/A	N/A
MG 5462	^58^	Mice	Via drinking water 1 × 10^9 CFU daily	56 days	C57BL/6 WT mice	Homeostasis	Increased frequency of CD3+, CD4+, CD8+T and NK1.1+ cells, and TNF-α production	N/A	N/A	N/A	N/A
ATCC PTA 6475	^59^	Mice	Oral gavage: 300uL 1×10^9 CFU/mL 3 times weekly Via drinking water: 1.5 × 10^8 CFU/mL	4 weeks	Female BALB/c mice in an ovari-ectomized-induced bone loss model	Homeostasis	Decreased CD4+ T-lymphocytes	N/A	Increased α-diversity; higher abundance of *Clostridiales* and lower abundance of *Bacteroidales* OTUs	N/A	N/A
No information provided	^60^	Mice	Oral gavage 6×10^7 CFU	Once	C57BL/6 GF	Homeostasis	Translocation to liver; microbial adaptation from mucosal to luminal niches	N/A	N/A	lacS/greA/ccpA triple mutation of *L. reuteri* facilitates translocation to and persistence at systemic sites (e.g. liver)	N/A
ATCC	^17^	Mice	Oral gavage 10^9 CFU daily	4 days	C57BL/6 GF Tet2-/- and Tet2+/+; Hemato-poietic-specific Tet2-/- with AIH-like pathology or symptom free; CD8 T Cells specific Tet2-/- in conca-navalin A-induced hepatitis; CCL4-induced hepatic injury	Autoimmunity	Translocation to liver-induced AIH in CD8 T cell specific Tet2-/- mice via secretion of I3A; induced Tc1 cell differentiation by decreased sensitivity to exogenous IFN-γ; increased activation of STAT1	Increase	Hematopoietic-specific Tet2-/- had increased microbial diversity including *L. reuteri*, *L. johnsonii*, *Enterococcus faecalis*, and *Escherichia coli* compared to symptom free littermates	peg257 mutants modestly predominated in the feces	N/A
No information provided	^61^	Mice	Oral gavage 10^9 CFU	Once	PWD/PhJ or C57BL/6 GF mice in MOG35–55/CFA EAE model	Autoimmunity	Produced tryptophan-derived metabolites and competed with host kynurenine pathway	Increase	N/A	*L. reuteri* tryptophan-derived metabolites activated the AhR and enhanced T cell production of IL-17	N/A
DYNDL22M62	^62^	Mice	Oral gavage 10^9 CFU daily	3 weeks	C57BL/6 WT mice in DNFB AD model	Autoimmunity	Alleviated swelling in ear; reduced serum IgE, TSLP, and IL-4 levels; activated AhR via increased levels of indoleacetic acid and indole propionic acid levels in fecal samples	Decrease	Reduced Dubosiella and increased *Romboutsia*, *Erysipelotrichaceae*, *Peptostrepto-coccaceae*, *Akkermensiaceae*, *Lactobacillaceae* and *Bifidobacteriaceae* in gut	N/A	N/A
FN041/DSM 17,938	^63^	Mice	Oral gavage 10^9 CFU daily	10 days or 3 weeks	BALB/C mice in MC903/ OVA-induced AD model	Autoimmunity	Strain specific induction of CD4+CD25+Foxp3+ Treg proliferation in spleen; decreased plasma IL-4, and ear IL-33 and TSLP levels	Decrease	Increased abundance of *Akkermansia*, *Limosilactobacillus*, *Faecalibacterium* and *Bifidobacterium* levels in ileum	N/A	N/A
No information provided	^64^	Mice	Oral gavage 10^7 CFU daily	7 or 18 days	FVB/N mice in L-NAME/salt hyper-tension model and C57BL/6 mice in MOG35–55/CFA EAE model	Autoimmunity	Reduced systolic and normalized diastolic blood pressure in hypertension model; reduced TH17 cells in spleen and spinal cord in EAE model	Decrease	N/A	N/A	N/A
H4/LMG18238	^65^	Mice	Oral gavage amount not specified	Once	C57BL6/J WT, GF and transgenic 2D2 TCR mice in EAE model	Autoimmunity	Express UvrA, a protein that mimics the TCR-binding residues of MOG peptide fragment 40–48; Uvra can cross react and activate MOG-specific T cells and increase Ki67+CD4+ T cells, exacerbating symptoms	Increase	N/A	Express peptides (UvrA) that mimic the MOG-specific TCR signals	In combination with a *Erysipelo-trichaceae* strain increased pathogenicity of MOG-specific Th17 cells and induced demyelination
No information provided	^66^	Mice	Oral gavage 10^9 CFU	Once for GF and 3 weeks for other mice	C57BL/6 WT, GF or TLR7.1 Tg mice in imiquimod cream (LR7 agonist) lupus model	Autoimmunity	Translocation to systemic sites resulted in type 1 immune response by increased plasmacytoid dendritic cells in spleen and mesenteric lymph nodes, and increased type 1 interferon signaling; growth inhibited by resistant starches and fermentation to SCFAs (e.g. butyrate)	Increase	Resistant starch suppressed intestinal *L. reuteri* abundance and translocation	Resistant starch exerts beneficial effects in lupus-prone hosts through suppression of *L. reuteri* which promotes interferon pathways implicated in the pathogenesis of human autoimmunity	N/A
DSM 17,938	^67^	Mice	Oral gavage 10^7 CFU daily	1 or 2 weeks	C57BL/6 WT or B.6Cg-Foxp3Sf/J mice (scurfy) in Fox3p+ Treg cell deficient autoimmunity model	Autoimmunity	Reduced IFN-y and IL-4 producing CD4+T cells in spleen and MLNs and levels of IFNy and IL-4 in plasma; metabolite inosine reduced TH1/TH2 populations via interaction with A2A receptor	Decrease	Restored Shannon α-diversity; increased relative abundance of the phylum *Firmicutes* and genera *Lactobacillus* and *Oscillospira;* decreased phylum *Tenericutes* and genera *Bacteroides*	N/A	N/A
CNCM-I5022/CNCM-I5429	^40^	Mice	Oral gavage 10^9 CFU 6 times weekly	3 weeks	NOD/DQ8 celiac disease susceptible murine model	Autoimmunity	Produced AhR ligands that modulated gluten immunopathology; decreased intraepithelial lymphocyte counts in low-tryptophan diet; improved villus-to-crypt ratio	Decrease	N/A	N/A	N/A
ATCC PTA 6475	^68^	Mice	Via drinking water 3.5 × 10^5 CFU daily	1 or 10 months	CD-1 Swiss mice and MMTV-neu (HER2) FVB mice in mammary tumor model	Cancer	Increased in Foxp3+ Treg cells and IL-10 levels in lymph nodes; lowered levels of IL-17 in lymph tissue and serum; reduced mammary mast cells in a CD4+CD25+ Treg cell dependent manner; decreased nuclear NF-κB and c-Jun in neoplastic cells	Decrease	N/A	N/A	N/A
ATCC PTA 6475	^69^	Mice	Oral gavage 5 × 10^9 CFU daily prior to AOM injection and every 3 days after DSS treatment	7 days then 15 weeks	*Hdc*-/- BALB/c in AOM/DSS CRC model	Cancer	Increased abundance of bacterial histidine decarboxylase mRNA and histamine in intestine; decreased KC, IL-22, IL-6, IL-1α, TNF in colonic mucosa; induced maturation of circulating immature CD11b+Gr-1+ myeloid cells	Decrease	N/A	N/A	N/A
ATCC PTA 6475/PRB94	^70^	Mice	Oral gavage 0.2 mL of 2 × 10^9 CFU/mL	up to 35 days	C57BL/6J tamoxifen-regulated CDX2p-CreERT2 transgene targeting Apc/Tpr53 knockout and Kras G12D knock in human intestinal cancer lines (RKO, DLD1, HCT118 and SW480) and various other	Cancer	Lowered grade and reduced invasive lesions; increased ROS species; decreased Ki67 cells and increased cCASP3	Decrease	N/A	N/A	N/A
ATCC 23,272	^71^	Mice	Oral gavage 10^8 CFU every other day or ILA (0.1 mg kg-1)	4 weeks	*APC*min mice sponta-neous CRC model C57BL/6J colon organoid model human embryonic kidney (HEK-293) cell line	Cancer	Produced ILA via aromatic amino acid aminotransferase; downregulated *IL17α* expression and reduced CD4+IL-17A+ T cells; inhibited nuclear transcription factor (RORγt); activated AhR in epithelium and improved gut barrier integrity	Decrease	N/A	N/A	N/A
MG5346	^72^	Mice	Via drinking water heat-killed 10^9 CFU	19 days	BALB/c nude mice human gastric cancer MKN1 cell line	Cancer	anti-tumor effect; high cell cytotoxicity; induced mitochondrial-dependent apoptosis; increased expression levels of p-AKT, p53, Bax, cleaved caspase-9/3 and cPARP in tumor tissue	Decrease	N/A	N/A	N/A
No information provided	^73^	Mice	Oral gavage 2×10^8 CFU twice weekly	3 weeks	C57BL/6 mice in a DEN/CCL4-induced HCC model	Cancer	produced acetate; inhibited IL-17A-producing ILC3s via Sox13 and reduced tumor burden	Decrease	N/A	N/A	N/A
ATCC BAA-2837	^16^	Mice	Oral gavage 10^9 CFU Daily or I3A at 20 or 40 mg/kg intratumoral: 2 × 10^7 CFU 200ug/mL	15–35 days	C57BL/6 mice in B16-F0 or YUMM1.7 melanoma, MC38 adeno-carcinoma, MMTV-PyMT breast cancer models serum samples of advanced melanoma patients	Cancer	Intratumoral presences promoted antitumor Tc1 immunity, suppressed tumor growth and increased survival; produced I3A which was required and sufficient to enhance Tc1 differentiation that depended on AhR activation within CD8 T cells	Decrease	Reduced gut microbial diversity and enrichment of *L. reuteri*; translocation to tumor, liver, spleen and mesenteric lymph nodes not taxa specific	Dietary tryptophan was derived I3A promoted tumor suppression; high levels of sera I3A correlated with prolonged progression-free survival in human cancer patients, overall survival and immune checkpoint inhibitor therapy response	N/A
#5529 refers to oligotype	^18^	Mice	N/A	N/A	C57BL/6 GF or hematopoietic-specific, intestinal specific and myeloid specific Tet2-/- mice in DSS colitis model	Cancer	Enriched in the jejunum of Tet2-/- mice; microbial cell wall components that bind to TLR2 sufficient to promote PMP independent of intestinal barrier dysfunction	Increase	N/A	Tlr2 agonist was sufficient to induce myeloid leukemia phenotype in an IL-6 dependent manner	N/A
***In Vitro*** **Models**											
MG5462MG4722MG5149	^74^	Cells	5 mg/mL cell free supernatant	24 hours	Murine macro phage RAW 264.7 cell line	Homeostasis	N/A	N/A	N/A	N/A	N/A
DSM 17,938	^75^	Cells	5% cell-free supernatant in MRS broth	24 hours	Retinoic acid- driven mucosal-like or monocyte derived dendritic cells from peripheral blood mono-nuclear cells of healthy donors	Homeostasis	N/A	N/A	N/A	N/A	N/A
ATCC PTA 6475, 5289, ATCC 55,730, CF48-3A	^76^	Cells	Biofilm cultures: 5% v/v Bacteria: diluted 1:100 in MRS broth	16 to 18 hours	Human mono-cytoid (THP-1) cell line	Homeostasis	N/A	N/A	N/A	N/A	N/A
ATCC PTA 6475	^77^	Cells	Conditioned media with histamine: 5% v/v Bacteria: diluted at 1:50 in MRS broth	16 to 18 hours	Human mono-cytoid (THP-1) cell line	Homeostasis	N/A	N/A	N/A	Bacterial histamine activated H2 receptor resulting in suppression of TNF production	N/A
PTA5_F13	^39^	Cells	10^7 CFU were added to each reactor daily	8 days	Novel polyfermentor intestinal model chicken cecal fermentation model	Homeostasis	N/A	N/A	Enriched *Clostridium innocuum* ASV075, *Lactobacillus* ASV016, *Monoglobus* ASV067 and *Faecalibacterium* UBA1819 AASV045; Decreased *Escherichia/Shigella*; in combination with glycerol protects against *Enterobacteriaceae* growth	N/A	N/A
PTCC 1655	^78^	Cells	1:10, 1:100 or 1:100 ratio	24–72 hours	Gastric adeno-carcinoma epithelial cell line (AGS)	Cancer	N/A	N/A	N/A	N/A	N/A
ATCC PTA 6475	^79^	Cells	10^9 cells mL-1	up to 24 hours	Human myeloid leukemia-derived cells (KBM-5) and human embryonic kidney cells (A293) cell lines	Cancer	N/A	N/A	N/A	N/A	N/A

In addition to EPS and BSH, *L. reuteri* releases several immunomodulatory metabolites, including reuterin, indole-derivates, adenosine, and histamine, which have been found to suppress intestinal inflammation.^84^ The metabolomes of *L. reuteri* can vary from strain to strain: *L*. *reuteri* ATCC PTA 6475 is mainly associated with histone production, and *L. reuteri DSM 1793*8 and its derived strain *L. reuteri* BG-R46 are typically known to produce adenosine.^85^ These strain-specific differences in their metabolite production can impact the ability of *L. reuteri* to influence host immunity. The *L. reuteri*-derived tryptophan catabolite indole-3-lactic acid (ILA) induces an expansion of CD4^+^ CD8αα^+^ double-positive intraepithelial lymphocytes by activating the AhR and subsequently suppressing the transcription factor Thpok in CD4 T cells.^57^ Both *L. reuteri*-derived ILA and a tryptophan-enriched diet potently induced this regulatory mucosal cell population. Notably, this study provided a mechanism of how the interplay of a probiotic bacterium (*L. reuteri*) and diet (tryptophan) can reprogram mucosal CD4 T cell immunity to potentially protect from enteric pathology.^57^ Indole-3-aldehyde (I3A), another dietary tryptophan catabolite produced by *L. reuteri*, can enhance type 3 innate lymphoid cells (ILC3s) and their production of IL-22, IL-10, and antimicrobial peptides that decrease intestinal inflammation.^86^ Many tryptophan catabolites direct their effects on immune cells through activation of AhR, a ubiquitous host cell transcription factor.^87^ Given the ubiquitous expression of AhR across immune cell types, tryptophan catabolites produced by *L. reuteri* effect a wide range of host immune cells, which is reviewed elsewhere.^88^ Histamine production by *L. reuteri* can directly suppress inflammation by dampening TNF production,^77^ or by driving myeloid plasmacytoid dendritic cells (DCs) to produce IL-10 instead of inflammatory cytokines upon LPS exposure.^89^ Secretory IgA (sIgA) serves as the first line of defense in protecting the intestinal epithelium from enteric toxins and pathogenic microorganisms.^90–92^ Although the mechanism is not fully revealed, studies show that *L. reuteri* stimulates sIgA production by expanding pre-germinal center (GC)-like and GC-like B cells in *Peyer*’s patches, potentially in a vitamin A-dependent manner.^55,93^ It has also been reported that *L. reuteri* can increase salivary IgA, but the evidence is contradictory and suggests an effect that is highly context- and strain-dependent.^43–45^ The differing conclusions may also be due to the quickly changing dynamics of the oral microbiome.

Exploring the interactions between *L. reuteri* and existing clinical treatments, and even other commensals, while not a focus of this review, are critical, especially as *L. reuteri* is commonly used as a probiotic and a therapeutic intervention in many different diseases. Research studies examining potential combinatorial treatments with *L. reuteri* have been increasing, especially in combating the side effects of more hazardous treatments. *L. reuteri* has been shown to protect against chemotherapy-induced oral mucositis^94^ and cisplatin-induced renal inflammation when used alongside *Clostridium butyricum*.^95^

### *L.* *reuteri* translocates to systemic organs during homeostasis

While several mechanisms of how *L. reuteri* improves intestinal barrier integrity are described (see paragraph above), numerous reports highlight *L. reuteri*’s ability to translocate systemically. *L. reuteri* is detected in various body sites, including mesenteric lymph nodes, spleen, urinary tract, and liver during both homeostasis and disease.^16–18,32,60,66,96^ One explanation for migration is that *L. reuteri* can directly attach to IECs via their mucus-binding proteins (MUBs).^97^ MUBs are encoded by *L. reuteri*, but quantitative mucus adhesion can be strain-dependent, with mucus binding highly correlating with the presence of MUBs.^97^ Although the mechanisms of *L. reuteri* translocation have not been fully revealed, a recent study discovered that within-host evolution contributes to the acquisition of mutations in commensal bacteria such as *Enterococcus gallinarum* and *L. reuteri* that facilitate translocation to systemic sites during homeostasis.^60^ This study shows that despite the important observation that certain *de-novo* mutations facilitate the translocation of commensals such as *L. reuteri*, these mutations are not required for translocation to systemic sites. Additional studies by the Meisel lab^16–18^ (and M.M. unpublished observations) and others^32,98,99^ corroborate the phenomenon of bacterial translocation of commensals to systemic sites during homeostasis. However, whether homeostatic commensal translocation to systemic organs contributes to the maintenance of immune homeostasis or the susceptibility to diseases remains to be defined.

### *L.* *reuteri* immunomodulation in the context of autoimmunity

**Figure 2.**
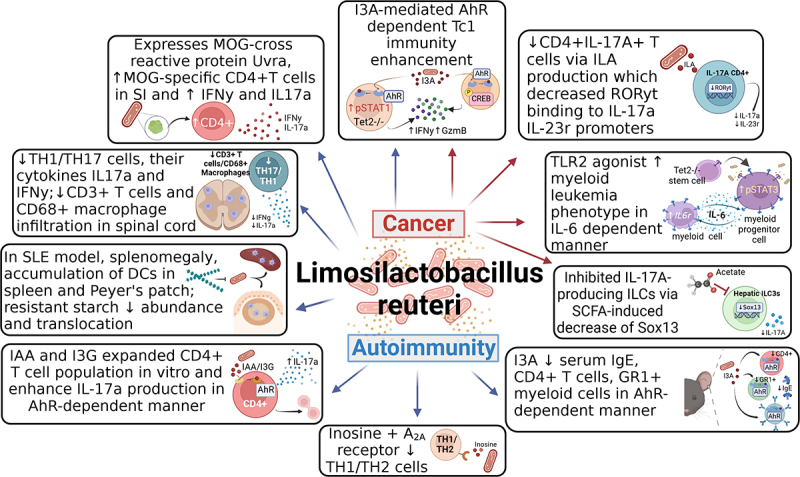
Role of *Limosilactobacillus reuteri* in the context of autoimmunity and cancer. *L. reuteri* is associated with both a protective and aggravating role in disease pathology. Strain-specific *L. reuteri* express MOG-cross reactive peptides which increase IL-17A cytokines and IFN-γ. Administration of *L. reuteri*, or its metabolites such as inosine, IAA or I3A, increases IL-17A producing TH17 cells, decreases TH1/TH2 population via interaction with the A_24_ receptor, and decreases serum Ig3. Depending on the cancer type, *L. reuteri* modulates cancer pathogenesis by increasing maturation of CD11b+Gr1+ myeloid cells or via TLR2-IL6 pathway promoting a myeloid leukemia phenotype. *L. reuteri*-derived metabolites influence disease progression. Specifically, I3A enhances Tc1 immunity via AhR activation in autoimmune and cancer disease models. Acetate, a short chain fatty acid, reduces IL-17A hepatic ILC3s through histone deacetylase inhibition. Diets supplemented with resistant starches decrease the proinflammatory effects of *L. reuteri* in autoimmune pathologies, decrease bacterial translocation, and reduce splenomegaly and accumulation of dendritic cells in spleen and Peyer’s patch. The summarized characteristics of this figure should not be considered as common characteristics of this species but rather possible functions of the species in context of homeostasis and systemic diseases.

Legend: A24, adenosine receptor 24; AhR, aryl hydrocarbon receptor; CREB, cyclic AMP response element binding protein; DC; dendritic cell; I3A, indole-3-aldehyde; I3G, indol-3-ylmethyl glucosinolate; IAA, Indole-3-acetic acid; IEC, intestinal epithelial cell; IgE, immunoglobin E; IL, interleukin; ILA, indole-3-lactic acid; ILC, innate lymphoid cell; INFy, Interferon gamma; ISC, intestinal stem cell; GzmB, Granzyme B; Lrg5, leucine-rich repeat containing G-protein-coupled receptor 5; MOG, myelin oligodendrocyte glycoprotein; SAA, serum amyloid A; SCFA, short chain fatty acid; sIgA, secretory immunoglobin A; SLE, systemic lupus erythematosus; Tc1, cytotoxic type 1; Tet, tet methylcytosine dioxygenase; TH, T-helper; T-TLR2, Toll-like receptor 2; TNF-α, tumor necrosis factor alpha; Treg, regulatory T cell.

The onset of autoimmune diseases is influenced by an interplay of a wide variety of factors, including genetics, environmental exposures, diet, and notably, the gut microbiota.^100,101^ *L. reuteri* exhibits a wide range of immunomodulatory functions in autoimmune disease, with studies indicating both protective and exacerbating roles for *L. reuteri* depending on disease model and context (Table 2 and Figure 2).

**Table 2.** Impact of *L. reuteri* colonization during gastrointestinal pathologies and infections.

Strain of *L. Reuteri*	PMID	Species	Condition/ treatment	Duration of treatment	Model	Pathology	*In vitro* effects	*In vivo* effects	Effect on disease severity	Effects on gut microbiome	Mechanisms	Comments
**Human Trails**												
NCIMB 30,242	114	Human	Orally administered 3×10^9 CFU/tablet daily (wk 1) and up to 9 × 10^9 CFU/tablet twice daily (wk 2–4)	4 weeks	Hypercholesterolemic individuals	Metabolic disorders	N/A	Increased circulating bile acid levels due to increased activation of FGF-19.	Decrease	Increased *Firmicutes* to *Bacteroidetes* ratio	N/A	
DSM 17,648	115	Human	Orally administered 4 tablets 5 × 10^9 CFU/tablet daily	14 days	*H. pylori*-positive patients	Enteric infection	Co-aggregation between *L. reuteri* and *H. pylori*	N/A	Decrease	Decreased *H. pylori* load	N/A	
ATCC 55,730	116	Human	Orally administered 4 tablets 10^8 CFU/tablet daily	4 weeks	*H. pylori*-positive patients	Enteric infection	N/A	N/A	Decrease	Decreased *H. pylori* load by 70%	N/A	
ATCC 55,730	117	Human	Orally administered 2 tablets 10^8 CFU/tablet daily	28 days	*H. pylori*-positive patients	Enteric infection	N/A	N/A	Decrease	Decreased *H. pylori* load	N/A	
No information provided	118	Human	Orally administered 10^8 CFU 3 times daily	21 days	*H. pylori*-positive patients	Enteric infection	N/A	N/A	Decrease	Increased eradication rate of *H. pylori* in patients	N/A	Patients were in eradication therapy for first 7 days
DSM 17,938/ATCC PTA 6475	119	Human	Orally administered 2×10^8 CFU 7 times daily	28 days	*H. pylori*-positive patients	Enteric infection	N/A	N/A	Decrease	Increased eradication rate of *H. pylori* in patients by 12.2%	N/A	Patients were in PPI therapy the whole time
DSM 17,938/ATCC PTA 6475	120	Human	Orally administered 2×10^8 CFU total daily	96 days	*H. pylori*-positive patients	Enteric infection	N/A	N/A	Decrease	Increased eradication rate of *H. pylori* in patients by 9%	N/A	Patients were in eradication treatment on days 29 to 35
DSM 17,938/ATCC PTA 6475	121	Human	Orally administered 2×10^8 CFU total daily	4 weeks	*H. pylori*-positive patients	Enteric infection	N/A	N/A	Decrease	Increased eradication rate of *H. pylori* in patients receiving eradication treatment by 8.6%	N/A	Patients were in eradication therapy for first 14 days
DSM 17,938/ATCC PTA 6475	122	Human	Orally administered 2×10^8 CFU/tablet twice daily	14 days	*H. pylori* -positive patients	Enteric infection	N/A	N/A	Decrease	Increased eradication rate of *H. pylori* in patients receiving eradication treatment by 8%	N/A	Patients were in PPI therapy for 14 days
ATCC 55,730	123	Human	Orally administered 10^8 CFU/tablet twice daily	4 weeks	Individuals with AAD	Enteric infection	N/A	Reduced frequency of diarrhea by 42.2%	Decrease	N/A	N/A	
DSM 17,938	124	Human	Orally administered 10^9 CFU daily	During hospi talization	Children with nosocomial diarrhea	Enteric infection	N/A	No impact of *L. Reuteri* administration observed	N/A	N/A	N/A	
DSM 17,938	125	Human	Orally administered 10^8 CFU daily	During hospitalization	Children with nosocomial diarrhea	Enteric infection	N/A	No impact of *L. Reuteri* administration observed	N/A	N/A	N/A	
**Animal Models**												
6475	126	Mice	Oral gavage 5 × 10^9 CFU daily	7 days	BALB/c, in TNBS colitis model	Intestinal inflammation	N/A	Enhanced ability to convert L-histidine to histamine via *hdcA*; microbial derived histamine activated H2R; decreased relative amounts of intestinal mucosal *Il6* and *Tnfα*	Decrease	N/A	Reduced colitis in a H2R-dependent manner	
100–23	51	Mice	Oral gavage or intravaginal administration 10^9 CFU	Once	C57BL/6 WT, BALB/c SCID or C3H/Orl GF mice in antibiotic treated DSS colitis model with *Candida albicans* injection	Intestinal inflammation / infection	N/A	Production of I3A-induced colonic AhR-dependent IL-22 secretion by NKp46+ NK cells and stomach mucosal ILC3s; restored antifungal resistance in GF mice	Decrease	N/A	Bacterial derived I3A-induced AhR-dependent IL-22 by NK and ILC3 cells	
R2LC	59	Mice	Oral gavage 10^8 CFU daily	7 days	C57BL/6 WT or CX3CR1-GFP/+ in DSS colitis model	Intestinal inflammation	N/A	Increased CD3-CD19+B220+ B cell population in *Peyer’s* patches; promoted bacterial defense by downregulation of *Sgpl1* and upregulation of *Lass5;* reduced levels of *TNF*α, *CXCL1, IL6* and *IL10;* increased IgA production via the Tfh-PD-1 pathway	Decrease	Preserved α-diversity; inhibited *Erysipelo-trichaceae* and *Escherichia/* *Shigella;* promoted *Bifido-bacteriaceae* and *Corio-bacteriaceae*	Increased IgA production via the Tfh-PD-1 pathway; increased B cells subset; protected against inflammation	
Lr 5454	93	Mice	Oral gavage 5 × 10^8 CFU daily	TNBS model: 6 days *C. rodentium* model: 14 days	C57BL/6 WT or GF, and BALB/c WT mice in TNBS or *C. rodentium* colitis model	Intestinal inflammation / infection	N/A	Decreased colonic gene expression of *Mip2, IL1*β, *IL6 and Tnfα*; promoted differentiation of CD4+CD25+ FoxP3+ Treg cells triggering *IL22* expression; increased intracellular IL-10 in CD4+ T cells; increased antimicrobial peptides gene expression of *mbd2*, *Reg3*β and *Reg3*γ in a NOD2-independent manner	Decrease	N/A	Promoted differentiation of CD4+CD25+ Foxp3+ Treg cells; increased intracellular cytokine IL-10 in CD4+T cells; increased *B-defensin-2*, *Reg3*γ and *Reg3*β gene expression in a NOD2-independent manner	
Used purified stress protein GroEL	127	Mice	Intrarectal administration: 1ng of protein daily	4 days	BALB/c mice in DSS colitis model Human PBMC macrophage isolate Ex-vivo human colon biopsy model	Intestinal inflammation	In human macrophages, inhibited LPS-induced pSTAT1 and promoted STAT6 and c-Myc expression; decreased TNF-α, IL-1β and IFN-γ and increased IL-10; decreased p-STAT1, caspase 3 and ROS in human colon explants	Reduced colitis symptoms and maintained higher number of intestinal crypts; reduced colon macrophages; increased IL-10 and IL-13 and decreased IFN-γ; may involve TLR4 and the non-canonical pathway	Decrease	N/A	Derived GroEL stress protein activated the production of IL-10 via TLR4, promoting M2-like macrophages, and inhibited development of M1-like macrophages	
D8	35	Mice	Oral gavage: 10^8 CFU Organoid: 10^6 CFU	Mice: 28 days Organoid: 48 hours	C57BL/6 WT mice in *C. rodentium* colitis model Mouse intestinal organoid model	Enteric infection	Increased intestinal epithelial proliferation partly via Wnt/β-catenin; increased mRNA expression of *c-Myc*, *cyclin*, *Ki67*, *Wnt3* and *Lrp5;* induced R-Spondin expression	Decreased intestinal TNF and IL-1β; modulated overactivation of the Wnt/β-catenin pathway; increased density of lysozyme+ Paneth cells; increased antimicrobial expression of *Defa1, Defa6* and *Lyz-1*.	Decrease	N/A	Increased expression of *Wnt3*, *Lrp5* which activated Wnt/B-catenin pathway; increased *Ki67*, *c-Myc* and *cyclin* which increased proliferation and epithelial repair	
FSCDJY33M3 FGSZY33L6 FCQHCL8L6	128	Mice	Oral gavage: amount not specified	14 days	C57/6N mice in alcohol-induced colitis model	Intestinal inflammation	N/A	Strain FSCDJY33M3 reduced mRNA levels of *TNFα*, *Il6*, *IL1β* and increased expression of *Muc2*, *Occludin* and *Claudin-3*	Decrease	Induced differences in β-diversity; Increased abundance of *Verruco-microbia* and *Eubacterium ruminatium*; decreased *Patesci-bacteria*	N/A	
DSM 17,938	94	Mice	Oral gavage: 10^6 CFU x g body wt −1 daily	5 days	C57BL/6 WT and B6/129-TLR2tm1kir/J TLR2-/- mice in cold exposure and hypoxia necrotizing enterocolitis model	Intestinal inflammation	N/A	Reduced colitis by 27%, increased percentage of CD44+CD4+ T cells; increased CD4+Fox3p+ Treg cells and CD103+ DCs via expression of CD80 and CD86; and decreased IL-1β and IFN-γ in ileal in WT not in TLR2-/-	Decrease	N/A	Regulated inflammation in a TLR2-dependent manner by activation of mucosal tolerogenic DCs which prime T cells into Tregs and reduced levels of IL-1β and IFN-γ in WT mice	
Post-biotics derived from *L. reuteri*	129	Mice	Oral gavage 30, 60 or 90 mg ml-1	23 days	C57BL/6J WT mice in alcohol-induced liver injury (NIAAA) model	Hepatic injury	N/A	Regulated intestinal FXR activated by bile acids; modulated FXR/SHP/SREBP-1c (potentially) ameliorating hepatic steatosis	Decrease	N/A	N/A	
**In Vitro Models**												
Exopolysaccharide from L26	130	Cells	100 ug/mL	4 hours	Enterotoxigenic *E. coli-*infected intestinal porcine enterocytes IPEC-J2 or monocyte-derived dendritic cell from healthy pigs	Enteric infection	Downregulated mRNA levels of *IL-8*, *TNF-α*, *IL-6*, *IL-1β*, *IL-12p35*, *TLR4*, *TLR5* and *MyD88*	N/A	N/A	N/A	N/A	
L26 (CCM 8616) Exopolysaccharide	43	Cells	Bacteria: multiplicity of infection 50:1 (bacteria: epithelial cell) Exopolysaccharide: 0.1 mg/mL	4 or 5 hours	Intestinal porcine enterocytes IPEC-J2 infected with *Salmonella Typhimurium*	Enteric infection	Increased mRNA levels of *IL-8* without infection and decreased levels with infection; decreased *IL-6*; increased *TNF-α* and *TGF-β*	N/A	N/A	N/A	N/A	
Exopoly Saccha ride from DSM 17,938 L26	131	Cells	10% v/v in MRS broth resulted in 5 g/L and 4.3 g/L	16 hours	Intestinal porcine epithelial (IPEC-1) cell line infected with enterotoxigenic *Escherichia coli*	Enteric infection	Increased expression of *IL-1β* by linear polysaccharide and upregulated mRNA levels of *NF-κB*, *IL-6* and *TNF-α*; inhibited *E. coli* adhesion and decreased IL-1β and IL-6	N/A	N/A	Inhibited adhesion of entero toxigenic *E. coli* to IPEC-1 Cells	N/A	

### Multiple sclerosis

Multiple sclerosis (MS) is a chronic T cell-mediated, progressive, neurodegenerative autoimmune disease. By performing shallow metagenomic sequencing, the international Multiple Sclerosis Microbiome Study (iMSMS) uncovered that MS patients display significant changes in fecal gut microbiome taxonomy and metabolic function when compared to healthy household controls.^101^ The role of *L. reuteri* in driving MS etiopathogenesis, however, is still uncertain. Many studies investigating the role of *L. reuteri* in MS disease models have conflicting results, making it difficult to classify *L. reuteri* as a symbiont or pathobiont in MS.

### Protective effect of *L.* *reuteri* in MS-like disease

One report showed that *L. reuteri* DSM 17,938 ameliorated the development of murine experimental autoimmune encephalomyelitis (EAE), a widely used animal model of MS, which is primarily mediated by T helper 17 (Th17) and Th1 cells.^102^ *L. reuteri* administration in this model significantly changed both the fecal microbiota alpha- and beta-diversity of mice developing EAE.^102^ *L. reuteri* administration reversed the EAE onset-induced changes to the microbiome, reducing the relative abundance of *Bacteroidetes* and restoring the relative abundance of *Proteobacteria* and *Deferribacteres*. Furthermore, an unbiased machine learning approach using genus-level relative abundance data uncovered that an operational taxonomic unit (OTU) matched to *L. reuteri* was the only effective OTU that distinguished control mice from mice susceptible to EAE.^102^ This suggests that *L. reuteri* may be an important driver in mediating protection from EAE. Indeed, upon *L. reuteri* administration, mice exhibited improved clinical EAE scoring and a decrease of Th17 and Th1 cells and their produced cytokines, IL-17A and interferon-γ (IFN-γ). Furthermore, *L. reuteri* significantly lowered CD3^+^ T cell and CD68^+^ macrophage infiltration into the spinal cord.^102^

Emerging research is etching out a key role for diet in commensal immunomodulation, as diet is critical in defining the metabolic repertoire of the microbiota.^103,104^ A key metabolic feature of *L. reuteri* is its ability to catabolize dietary tryptophan into immunomodulatory AhR ligands.^86^ A study discerning a role for *L. reuteri* in EAE suggests that *L. reuteri-*derived tryptophan catabolites may play a beneficial role in EAE.^64^ FVB/N mice fed a high salt diet (HSD) had a lower relative abundance of *Limosilactobacillus* as early as one day after diet administration and a significant reduction of total metabolite counts as determined by gas-chromatography-mass spectrometry, demonstrating the potent consequences of dietary interventions on the gut microbiota. Mice on a HSD displayed more severe pathogenesis as determined by clinical scoring, as well as significantly reduced fecal levels of the tryptophan catabolites ILA and indole-3-acetic acid (IAA). *L. reuteri* administration was able to reverse the HSD-induced effects, significantly lowering clinical scoring and the number of Th17 cells in the spleens and spinal cords of EAE mice. Suggestive of a role for *L. reuteri* metabolites in regulating Th17 immune response in EAE, ILA co-cultured with naïve CD4 T cells significantly reduced the percentage of IL-17A producing cells that developed under Th17-polarizing conditions in a dose-dependent manner. A small clinical pilot study also indicated that these results may be clinically translatable. Healthy volunteers ingesting 6 g of extra sodium chloride daily for 14 days saw a decrease in *Lactobacilli* abundance and an increased percentage of proinflammatory IL-17A and TNF-α producing CD4^+^ T cells in the peripheral blood.^64^

### *L.* *reuteri* aggravates MS-like pathology

Contrary to the previously mentioned study signifying a role for IAA in EAE suppression, another study found that in a different dietary context, *L. reuteri*-derived tryptophan catabolites can exacerbate inflammation in an EAE model.^61^ A diet high in tryptophan given to *L. reuteri*-colonized mice increased CD4^+^ IL-17A and IFN-γ producing cells and CD8^+^ T cells. *L. reuteri* isolates were enriched for high affinity classes of tryptophan catabolizing enzyme aromatic amino acid aminotransferase (ArAT). Unlike other *Limosilactobacillus* genomes, only *L. reuteri* isolates encoded aliphatic amidase E (AmiE), an enzyme that converts indole-3-acetamide into IAA. When *L. reuteri* was cultured in brain-heart infusion media supplemented with tryptophan, *L. reuteri* was able to increase concentrations of IAA, indole-3-lactate, tryptamine, indole-3-glyoxylic acid (I3G), and I3A. Metabolomics of serum from *L. reuteri*-colonized mice and *L. reuteri* monocultures revealed an increased presence of known and novel tryptophan-derived AhR ligands, including IAA, indole-3-glyoxylic acid, tryptamine, p-cresol, and diverse imidazole derivatives. Two of these metabolites, IAA and I3G, were able to expand CD4^+^ T cell populations in splenocyte cultures *in vitro* and enhance their IL-17A production in an AhR-dependent manner. Remarkably, mice on the diet low in tryptophan had significantly lower clinical scores, almost to the extent of complete disease prevention.^61^

Understanding the strain-specific effects of probiotics is critical for effective modulation of health outcomes.^4^ A study highlighted *L. reuteri*’s strain-specific effect in EAE etiopathogenesis by showing that one OTU, corresponding to *L. reuteri* strains H4 and LMG 18,238, activated autoreactive myelin oligodendrocyte glycoprotein (MOG) T cells thereby promoting EAE pathology.^65^ The authors observed that induction of EAE triggers an expansion of MOG-specific CD4^+^ T cells in the lamina propria of the small intestine that produce large amounts of proinflammatory IFN-γ and IL-17A cytokines. Shotgun sequencing of small intestinal contents for mimicry peptides with sequences that matched MOG_40–48_, the TCR binding region for MOG, revealed that more than half of the candidate mimicry peptides found were *Limosilactobacillus*-derived. Three of these mimicry peptides were found to be specific to *L. reuteri* strains H4 and LMG 18,238, including UvrA. Albeit weakly, UvrA co-culture was able to increase activation levels of MOG_33–55_ specific 2D2 CD4^+^ T cells, demonstrated by increased expression of Ki67 and CD44.^65^ This study puts forward the notion that some, but not all, *L. reuteri* strains are pathogenic in EAE and further highlights the importance to mechanistically interrogate the strain-specific effects of *L. reuteri* in the context of MS.

Additionally, one study suggests that while *L. reuteri* alone could not worsen EAE clinical scoring, *L. reuteri* and an OTU matching *Allobaculum stercoricanis* DSM 13,633 can work synergistically to worsen EAE disease. Especially given that many probiotic regimens contain several commensals in addition to *L. reuteri*, the interactions between the *L. reuteri* and other bacteria species are an important avenue of investigation in the future.

Taken together, the differing conclusions of the effect of *L. reuteri* on EAE etiopathogenesis may be (**i**) strain-specific, (**ii**) disease-model-specific, or (**iii**) driven by diet-dependent changes to *L. reuteri*’s environment and metabolomic profile.

### Systemic lupus erythematosus (SLE)

Systemic lupus erythematosus (SLE) is a chronic-progressing, multi-organ autoinflammatory disease. While not species-specific to *L. reuteri*, *Lactobacilli* have been found to be enriched in feces of patients with SLE.^66^ Translocation of commensal bacteria can enhance systemic immune cell activation and thus worsen or even trigger autoimmunity in genetically susceptible individuals.^32,98,105^ This is especially clear in examining the effect of a resistant starch (RS) supplemented diet on the effects of *L. reuteri* in a mouse model of SLE-like disease. *L. reuteri* translocated to the mesenteric lymph nodes, liver, and spleens of TLR7.1 transgenic (Tg) mice.^66^ *L. reuteri* gavage in these mice led to splenomegaly, plasmacytoid dendritic cell (DC) accumulation in the spleen and in *Peyer’s* patches, and increased leukocyte recruitment to the kidneys. In wild-type (WT) C57BL/6 mice treated with imiquimod, *L. reuteri* worsened anemia and elevated gut permeability. Interestingly, the administration of a RS diet decreased *L. reuteri* abundance and translocation, likely due to improvement of the gut epithelial integrity as demonstrated by increased expression of markers such as *Muc2* and *Reg3γ*. Both, WT C57BL/6 mice treated with imiquimod and TLR7.1 Tg mice fed with RS had reduced organomegaly, systemic type I IFN, and lowered frequencies of splenic pDCs, Th17 cells, neutrophils, and activated CD44^+^ T cells. However, *L. johnsonii* was also able to translocate to gut-distal sites but did not significantly worsen SLE-like disease, suggesting that there is an additional mechanism beyond translocation that is inhibited by a RS diet, such as the production of a metabolite.^66^ Despite the differing disease model, obese WT mice fed a high fat, resistant starch diet (HF-RS) decreased cecal IL-17A concentration and increased ileal Reg3γ and cecal occludin, supporting findings of Zegarra-Ruiz et al.^66^ that a RS diet may strengthen barrier function in autoimmune-prone mice.^106^ In a separate study, overexpression of Reg3γ was found to decrease the number of mucosa-associated bacteria (such as *L*. reuteri) and reduce the instance of bacterial translocation, consequently protecting mice against liver inflammation in an alcohol-induced steatohepatitis model.^107^

### Autoimmune hepatitis (AIH)

Autoimmune hepatitis (AIH) is an autoinflammatory liver disorder that often becomes refractory to immunosuppressants – the only therapeutic option for AIH patients – and progresses to end-stage liver disease in the absence of treatment.^108^ Despite previous work suggesting genetic and environmental factors play a role in AIH development,^109^ mechanisms underlying disease initiation remain enigmatic due to the lack of suitable murine models. Microbiota dysbiosis is observed in human AIH patients and a requirement of the microbiota for the development of experimental AIH is observed.^110^ However, the mechanisms of how the microbiota impacts on AIH pathogenesis remains poorly understood. We recently described a novel model of AIH-like disease that displays key features of human AIH.^17^ Using this novel AIH-like disease model, we showed that lack of hematopoietic Tet methylcytosine dioxygenase 2 (*Tet2*^*ΔVAV*^), an epigenetic regulator associated with autoimmunity, results in the development of microbiota-dependent AIH-like pathology, accompanied by hepatic enrichment of AhR ligand-producing pathobionts and Tc1 cell immunity.^17^ *Tet2*^*ΔVAV*^ mice displayed a significant change in liver microbiome composition when compared to littermate controls and symptom free *Tet2*^*ΔVAV*^ mice, characterized by an expansion of AhR ligand producing commensals such as *L. reuteri* and an increased AhR metabolism^17^. Oral gavage of *L. reuteri* was sufficient to trigger AIH-like disease in *Tet2*^ΔVAV^ mice, profiled by an increase in hepatic AhR activity, hepatocyte damage, autoimmune AIH markers such as antinuclear antibodies (ANA) and hepatic Tc1 immunity. This injurious effect was lost without the ability of *L. reuteri* to produce tryptophan catabolite I3A and in mice that lack AhR expression within CD8 T cells. We identified that naive *Tet2* deficient CD8 T cells display a heightened sensitivity to extrinsic IFN-γ. Therefore, I3A-induced IFN-γ production -that occurs independent of *Tet2*- promoted a vicious feedforward cycle that led to AIH-like pathology in our model. Accordingly, we found that genetic *Ifng* ablation prevented the development of, and neutralization of IFN-γ reverted ongoing, AIH-like pathology in our model.^17^ However, more pre-clinical and clinical studies are warranted to explore the role of the liver microbiota in AIH.

### Other autoimmune pathologies

Oral administration of *L. reuteri* was linked with an improvement of rheumatoid arthritis^48^ and was found to impact on the disease course of human atopic dermatitis patients (AD)^49,50,111^ (see Table 2). In a dinitrofluorobenzene (DNFB)-induced AD-model, DNFB treatment lowered the concentration of microbial tryptophan catabolites, especially ILA and indole-3-propionic acid, and suppressed AhR expression.^62^ Both AhR expression and the expression of tryptophan catabolites were restored by *L. reuteri* strain DYNDL22M62. *L. reuteri* strain DYNDL22M62 most significantly reduced ear swelling compared to strains FSDLZ12M1, GLDZ105 and FWXBH12M3. Additionally, *L. reuteri* strain DYNDL22M62 was the only strain able to suppress thymic stromal lymphopoietin (TSLP), as well as immunoglobin E (IgE), IL-4 and IL-5 alongside strain FSDLZ12M1.^62^ While this study does not address the requirement or sufficiency of tryptophan catabolites or AhR activation in improvement of clinical AD symptoms, another report corroborated that the ability of tryptophan catabolites to attenuate AD-like pathology is AhR dependent.^112^ Using an MC903 model of AD, the authors demonstrated that the tryptophan catabolite I3A was able to significantly reduce ear thickness, IgE levels, and CD4^+^ T cell and Gr1^+^ myeloid cell ear infiltrates, but this effect was abrogated in AhR-deficient mice. Mechanistically, I3A activated AhR, which then bound to the TSLP promoter to inhibit the production of TSLP in keratinocytes.^112^ Taking both studies into consideration, *L. reuteri* may play a protective role in AD through AhR activation mediated by its metabolites, but the effects differ appreciably from strain to strain.

Generally, *L. reuteri* mediates protection in autoimmune diseases through its ability to promote Treg cell differentiation. *L. reuteri* 17938 and 5454-derived supernatant increased the production of Foxp3^+^ Treg cells through activation of CD103^+^ DCs.^75,113^ *L. reuteri* 17938 also activated DCs through toll-like receptor 2 (TLR2) to expand Tregs in a model of necrotizing enterocolitis.^114^ In addition to increasing Treg populations, *L. reuteri* may combat autoimmunity in Treg-deficient disease models by decreasing inflammation. Colonization of *L. reuteri* can shift the microbiome composition, and can increase the production of certain metabolites, such as adenosine and inosine,^85^ by the gut microbiota in mice with Treg deficiency.^115^ In scurfy mice, a model of Treg deficiency, either *L. reuteri* or inosine alone was able to reduce Th1/Th2 cell populations and lower inflammation in Treg-deficient disorders through interactions with the adenosine A_2A_ receptor.^67^

### *L.* *reuteri* immunomodulation in the context of cancer

Cancer patients are increasingly interested in using probiotics to augment health:^13^ a recent study found almost half of its cohort of advanced melanoma patients initiating ICI treatment self-administered probiotics.^116^ *L. reuteri* has been highlighted for its ability to modulate antitumor immunity at both intestinal and gut-distal tumors, due its ability to translocate and produce highly immunomodulatory metabolites (Table 2 and Figure 2).

### Anti-tumor function of *L.* *reuteri* – cancer that arises at intestinal sites

Native to the gut, *L. reuteri* colonization and its metabolite production impacts the development and progression of colorectal cancer (CRC).^117^ Much of *L. reuteri*’s impact on gastrointestinal cancers is preventative, through homeostatic functions such as protecting the intestinal barrier, lowering chronic inflammation, and ameliorating infection by pathogenic bacteria that can drive cancer development.^118^ In an azoxymethane-dextran sodium sulfate model of CRC, *L. reuteri* significantly decreased the relative abundance of CD11b^+^ Gr1^+^ myeloid cells compared with mice that did not receive exogenous *Lactobacilli*, possibly playing a role in the prevention of colitis-associated cancer.^69^ *L. reuteri* can also lower tumor burden after cancer initiation in gastrointestinal cancers. ILA, a tryptophan catabolite produced by *L. reuteri*, downregulated IL-17 signaling and mediated the chemo-preventative effect of statins in murine CRC.^71^ Both *L. reuteri* and ILA gavage reduced tumor burden in a CRC murine cancer model. *Ex vivo* co-culture of naïve CD4^+^ T cells and ILA markedly reduced the percentage of IL-17A CD4^+^ T cells in a dose dependent manner. The reduction in Th17 response was mediated by the ability of ILA to decrease the binding of master transcription factor RORγt to *IL-17a* and *IL-23 r* promoters. Another *L. reuteri*-derived metabolite, histamine, was associated with survival in CRC patients.^69^ Patients with high expression of *HDC*, a gene encoding the key enzyme for histamine generation, had significantly higher survival. In a pre-clinical, chemically induced CRC model, *L. reuteri* was able to reduce tumor node abundance and overall tumor size. *HdcA* mutant *L. reuteri* that cannot produce histamine was less protective against CRC tumor burden, but still more protective compared to the control group. These findings importantly imply that the protective ability of *L. reuteri* is partially, but not completely, dependent on its histamine production.^69^ Reuterin, a *L. reuteri* metabolite most notable for its antimicrobial properties, was able to inhibit the proliferation and increase the apoptosis of colon cancer cells by increasing intracellular reactive oxygen species (ROS).^70^ Global transcriptomic analysis of CRC-cells revealed that reuterin upregulates genes in the oxidative stress pathway. The induction of apoptosis was specific to CRC cell lines HCT116, SW480, DLD1, and RKO, but was absent in primary cell lines, implying a tumor-specific effect.^70^ Another study indicated that heat-killed *L. reuteri* induced apoptosis through Akt-p53 dependent mitochondrial apoptosis in a MKN1 human gastric cancer cell line.^72^ Given that non-metabolically active *L. reuteri* can induce apoptosis in a tumor-specific manner, it is likely that there is a cellular component mediating tumor cell death.

### Anti-tumor function of *L.* *reuteri* – cancer that arises at extra-intestinal sites

Reinvigorating antitumor immunity by immune checkpoint inhibitor (ICI) treatment is a core component of cancer therapy that has shown unprecedented efficacy. However, only a fraction of cancer patients respond to ICI treatment.^104^ Approaches that further potentiate antitumor immunity are urgently needed to boost ICI efficacy. It is well accepted that the microbiome significantly impacts on anti-tumor immunity, as well as ICI-responsiveness.^104^ Still, the impact of probiotics on ICI responsiveness in cancer patients, as well as the mechanisms that drive these effects, remain poorly understood. In a recent study of our laboratory, we set out to test whether some of the most frequently used probiotics impact preclinical B16 melanoma outgrowth. While we observed that several probiotics restrained tumor outgrowth, *L. reuteri* displayed the most potent ability to restrain tumor growth and enhance antitumor Tc1 immunity.^16^ Furthermore, *L. reuteri* potentiated both αPD-L1 and αCTLA-4 immunotherapy. The antitumoral effect of *L. reuteri* extended to ICI-resistant BRAF^V600E^ melanoma, MC38 adenocarcinoma, and MMTV-PyMT breast cancer models. Accumulating evidence suggests the presence of a tumor microbiome in gut-distal cancer, and live bacteria have been recovered from breast and pancreatic patient tumors.^18,119–122^ However, it remained unaddressed whether intratumoral bacteria in gut-distal tumors are passive inhabitants or active participants that impact tumor development. By deploying a novel culturomics approach^123^ we uncovered that *L. reuteri* translocates to and persists in gut-distal tumors. Further investigation demonstrated that viable intratumoral *L. reuteri* was critical for the restraint of tumor growth, indicating that both the translocation and metabolic activity of *L. reuteri* was necessary for its antitumor potential. *L. reuteri*-derived I3A enhanced Tc1 immunity by activating AhR in CD8 T cells, leading to the significantly increased production of IFN-γ and expression of the Tc1-regulating transcription factor *Blimp1* by CD8 T cells. Remarkably, antitumoral immune responses in response to *L. reuteri* were local to the tumor, and could be recapitulated by intratumoral injections of I3A. We also identified a potential role of I3A in promoting ICI response and survival in melanoma patients. The enhancement of antitumoral Tc1 immunity by *L. reuteri* was dependent on dietary tryptophan, highlighting a key role of diet in probiotic immunomodulation.^16^ A recent randomized, controlled trial found that healthy volunteers that consumed 3 g of l-tryptophan supplements a day for 2 weeks had substantially increased AhR activation (by reporter cell assay) and production of tryptophan catabolites, including I3A.^124^ Additionally, participants did not suffer any abdominal or psychological symptoms while on a high tryptophan diet, suggesting that dietary tryptophan supplementation may be able to be successfully used alongside *L. reuteri* in future clinical trials.

A growing number of studies indicate that *L. reuteri* produces several metabolites with antitumor potential. Another *L. reuteri* produced metabolite, the SCFA acetate, was able to reduce tumor burden in a murine hepatocellular carcinoma (HCC) model by inhibiting IL-17A production.^73^ *L. reuteri* administration decreased overall IL-17A levels in serum and selectively reduced the IL-17A production of hepatic ILC3s, but not thymic or splenic cells. Acetate co-culture alone was able to reduce the percentage of IL-17A producing hepatic ILC3s through histone deacetylase (HDAC) inhibition. On a transcriptional level, acetate significantly decreased *Sox13* binding to the *IL-17a* promoter inhibiting IL-17A transcription. Acetate acted synergistically with αPD-1 ICI treatment to decrease tumor number and ILC3 production of IL-17A.^73^ While these studies highlight the ability of *L. reuteri* to suppress tumor growth, they also are highly suggestive that *L. reuteri* may be able to strengthen current treatment regimens being used in the clinic, such as ICI therapy and chemotherapy.

A report identified that *L. reuteri* administration significantly reduced tumor outgrowth of westernized diet-induced mammary tumors in Swiss mice. The protective effect of consuming *L. reuteri* occurred in a CD25 cell-dependent manner. Moreover, *L. reuteri*-treated donor CD4^+^ CD45RB^lo^ CD25^+^ immune cells were sufficient for the suppression of mammary tumors upon transfer into untreated recipient HER2/neu mutant mice.^68^

### Pro-tumor function of *L.* *reuteri*

Most studies centered around the role of *L. reuteri* in cancer show its ability to positively impact patient outcomes and enhance antitumor immunity, but there is some evidence that it may create tumorigenic environments in the context of certain genetic susceptibilities. In the absence of hematopoietic *Tet2*, we demonstrated that translocating *L. reuteri* in the circulatory system can act as a general TLR2 agonist, causing IL-6 receptor-overexpressing granulocyte-myeloid progenitor cells to differentiate into IL-6-producing CD11b^+^ Gr1^+^ myeloid cells.^18^ This continuous cycle of inflammation led to the development of pre-leukemic myeloproliferation, potentially setting the stage for the development of overt leukemia.

## Conclusion and future directions

**Figure 3.**
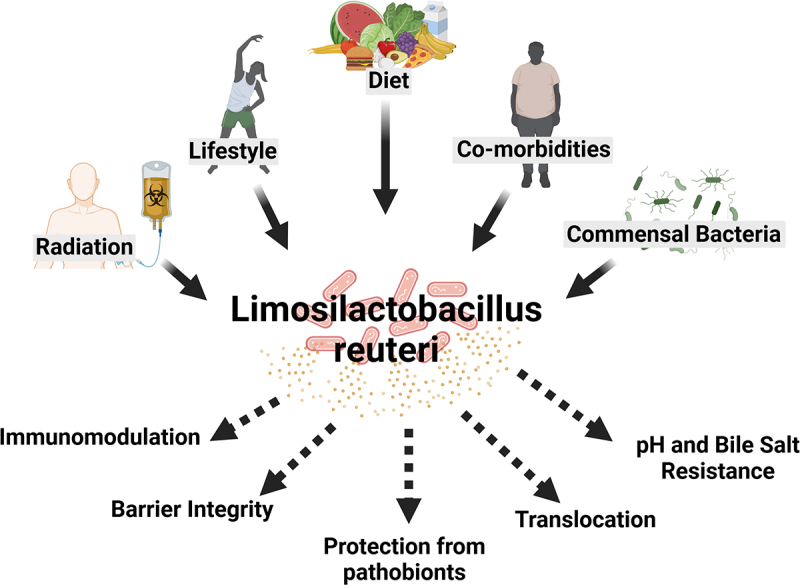
Environmental factors modulating microbe-host interactions in context of health and disease. *L. reuteri* is modulated by various environmental factors including lifestyle, diet, co-morbidities, presences of other commensal bacteria and exposure to radiation. In turn, *L. reuteri* and its metabolites have multiple impacts on the host via immunomodulation, intestinal barrier integrity, protection from pathobionts, translocation to extra-intestinal sites and resistances to intestinal pH and bile salts.

Despite being a widely consumed probiotic commonly found in many foods, *L. reuteri* has the remarkable ability to influence the course of disease. *L. reuteri* has revealed itself as a highly immunomodulatory probiotic bacterium, modulating local and systemic immune responses during steady state and complex pathologies, including intestinal inflammatory disorders, autoimmunity, and cancer. While the impact of *L. reuteri* in the context of gastrointestinal pathology is better understood,^15^ we are just beginning to uncover mechanisms of how *L. reuteri* tunes systemic immunity during homeostasis and in complex diseases such as autoimmunity and cancer (Figures 1 and 2). Previous studies demonstrate that the context (type of disease (e.g. autoimmunity, cancer) or environmental factors (e.g. diet) dictates whether *L. reuteri* behaves as a symbiont or a pathobiont. However, more mechanistic studies are required to better understand which and how environmental factor(s) modulate the host-immunomodulatory potential of *L. reuteri* (Figure 3).

One critical future area of focus involves understanding the strain-dependent effects of *L. reuteri*, as different strains can have widely differing roles on immunomodulation, as referenced throughout this review. Both strain-dependent effects as well as differing efficiencies of *L. reuteri* colonization may explain some of the conflicting results reported when examining the health impacts of *L. reuteri.*

The ability of *L. reuteri* to translocate has emerged only recently, yet many studies discussed here suggest the importance of translocation for *L. reuteri*’s effect on host immunity. The mechanisms on how *L. reuteri* translocates to systemic sites and the ability of homeostatic translocation to impact immunity are still not fully understood and require further mechanistic interrogation. A better understanding of *L. reuteri’s* translocation may also shed light on how other commensals modulate immunotherapies. This may be especially important in cancer contexts, given that the efficacy of cancer immunotherapy is highly linked with the presence of a cancer microbiome.^125^

One key mechanism by which *L. reuteri* modulates immune responses both during homeostasis and in complex diseases such as autoimmunity and cancer is through its produced metabolites. With this observation comes the exciting new question of how we can orchestrate the *L. reuteri* metabolome to drive certain immune responses. One way to tune the metabolic output of the gut microbiota, including commensals such as *L. reuteri*, is via the diet. There is emerging evidence that demonstrate that dietary changes be able to impact the effects of commensals on systemic immunity.^16,61,66,126^

It will be important in future studies to interrogate how we can use diet as a tool to precisely and selectively modulate microbial metabolic pathways in *L. reuteri* to tailor immunity to our needs. Unraveling the intricate and diverse relationships between host nutrition, the microbiome, and the metabolome will largely be driven by multi-omics approaches, including metagenomics, metaproteomics, culturomics, and metabolomics, in combination with mechanistic studies. On a clinical level, precision dietary inventions paired with microbiota-based therapeutics (e.g. probiotics, FMTs) will allow clinicians to calibrate the microbial metabolome for each patient.

Decades of studies discerning the numerous ways *L. reuteri* impacts human health outcomes now opens doors for new research to translate *L. reuteri* effectively into the clinic and sets up a foundation to unlock the immunomodulating mechanisms of other probiotic bacteria.
